# Participation in medical activities beyond standard consultations by Swiss general practitioners: a cross-sectional study

**DOI:** 10.1186/s12875-018-0738-1

**Published:** 2018-05-03

**Authors:** Julian Jakob, Christine Cohidon, Jacques Cornuz, Kevin Selby

**Affiliations:** 1 Department of Ambulatory Care and Community Medicine, Lausanne University, Rue du Bugnon 44, 1011 Lausanne, Switzerland; 2 Institute of Primary Health Care (BIHAM), University of Bern, Bern, Switzerland; 3 Institute of Family Medicine, Department of ambulatory care and community medicine, University of Lausanne, Lausanne, Switzerland; 4 Kaiser Permanente Division of Research, 2000 Broadway, Oakland, USA; 5 Policlinique Médicale Universitaire, Rue du Bugnon 44, CH-1011 Lausanne, Switzerland

**Keywords:** Primary care, Community medicine, Rural area, Complementary activities, Cross-sectional

## Abstract

**Background:**

Few data exist to support the observation that general practitioners (GPs) occupy many important positions in our communities or to characterize which GPs devote more of their time to such activities. We sought to characterize community-based complementary medical activities performed by GPs in the canton Vaud, Switzerland.

**Methods:**

All GPs in a region were invited to participate in a cross-sectional study (*n* = 600) examining engagement in complementary activities beyond standard ambulatory consultations. Categories included teaching, care giving in specific structures, roles as medical experts or company doctors, community care giving, and others completed by the GP. GPs were asked the number of hours devoted monthly to each activity and whether or not they are remunerated for this work.

**Results:**

One hundred and sixty-eight GPs responded (28%), with 149 (92%) reporting that they were engaged in at least one activity beyond their in-office consultations, including 117 (72%) in community care-giving (ex: care for addictions or refugees). Altogether, GPs spend on average 5.8 h a week on these activities. One-hundred and twenty-three GPs (82%) were remunerated for at least one of their complementary engagements. Predictors of participation in a larger number of complementary activities were working in a rural area (IRR 1.29, 95% CI 1.05 to 1.57) and having a higher weekly workload (IRR 1.01 for each additional hour, 95% CI 1.01 to 1.02).

**Conclusion:**

The vast majority of GPs engage in activities beyond their standard clinic tasks and they are typically reimbursed. GPs in rural areas and those who work more hours per week are more likely to engage in complementary activities.

**Electronic supplementary material:**

The online version of this article (10.1186/s12875-018-0738-1) contains supplementary material, which is available to authorized users.

## Background

A shortage of general practitioners (GPs) has been predicted for the coming years [1], due to the rising demand of health care services, restrictions on incoming medical school students (*numerus clausus*) and not enough young physicians choosing primary care specialties [2]. Beyond its impact on basic medical care, this shortage could cause an inadequate supply of GPs for various community activities, and rural areas may be affected most [3]. We know anecdotally that besides the *standard* activities provided by every GP, defined as traditional in-office consultations with patients and associated administrative tasks, medical doctors often occupy additional, *complementary* functions. Acting as company doctors, providing care in nursing homes, or insuring the education of students and residents appear to be most common [4]. However, rising demand for complementary community activities could exacerbate the shortage of GPs available for standard consultations [5]. On the other hand, GPs may find complementary activities desirable because they provide variety, stable income, and predictable hours, thus increasing the desirability of being a GP and potentially attenuating the GP shortage [6].

Currently, there are few data focusing on complementary activities performed by GPs outside the exam room. Cohidon et al. described a clear increase in the proportion of Swiss physicians who perform paid complementary activities, such as teaching, being engaged in a nursing home or involved in private enterprises besides the standard in-office health care delivery, from 28% in 1993 to 66% in 2012 (*p* < 0.001) [7]. Thirty-nine percent of GPs in the US report being engaged in voluntary medical activities [8], while 90% of GPs consider public roles important [9]. Dimensions of community engagement to improve medical care delivery in variable settings have been proposed [10]. However, these studies used narrow definitions of complementary activities, did not specify participation rates and time spent on each type of activity, and did not specifically study associations between GP characteristics and practice location with engagement in these activities. This information could have important policy implications and be used to attract attention to the important role GPs play in our community.

The goal of this study was to characterize and quantify complementary medical activities performed by GPs in the canton Vaud, Switzerland.

## Methods

### Setting and design

We performed a cross-sectional study of GPs in the canton of Vaud, Switzerland during the summer of 2016, first using paper questionnaires during a continuing medical education course, and later an online form distributed by email. GPs were explicitly informed on the title page of the online form to fill it only if they had not previously participated in the study during a continuing medical education course. This questionnaire had been developed with consultation of authors who had published studies about similar primary care topics [7]. Primary care in Switzerland is formed by a combination of physicians licensed in General Internal Medicine and General Medicine, referred to jointly here as general practitioners (GPs). Vaud is the third largest Swiss canton, with over 750,000 inhabitants, mostly French-speaking. Members of *Médecins de famille Vaud*, a professional association representing nearly all GPs in the canton, were invited to participate (*n* = 600) between June and August 2016. Paediatricians were not included. We used paper questionnaires during continuing medical education courses including around 100 physicians in this time period, followed by a mass email to all members. A reminder was sent one month after the first email.

### Survey instrument

We defined *standard* activities done by every GP as in-office consultations, completed by documentation of the visit and associated administrative tasks only. All medical activities that are not performed routinely by every physician and sometimes even require a special license, such as teaching (2 questions, pre- and post-graduate), care giving in specific structures (5 questions, i.e. schools and nursing homes), engagement as medical expert or company doctor (2 questions with 5 sub-items, i.e. insurance companies), community care giving (6 questions, i.e. supporting patients suffering from addictions, sporting events) and others as specified by the GP itself, were considered as *complementary* activities. In addition to Cohidon et al.’s definition of complementary paid activities, we included non-remunerated activities, but did not ask about office management and health care coordination engagements as these are part of a GP’s daily routine [7]. For each complementary activity, the GPs were asked the mean number of hours monthly spent on it, averaged over a year. GPs were asked to answer whether they receive financial compensation, though the amount or nature of such a financial compensation was not of interest in this study. The end of the questionnaire included demographic questions such as physician gender, year of birth and graduation, office size and location, country in which GPs completed their medical education, whether they belong to a medical network and average hours spent on standard in-office consultations. The French-language questionnaire and the resulting dataset are available upon request.

### Statistical methods

Descriptive statistics were presented using frequencies and means with standard deviations where appropriate. Activities left blank on otherwise complete questionnaires were considered as non-engagement in the complementary activity in question. A Poisson regression model was built on the primary outcome of the number of activities each GP is engaged in. Provider (gender, age, years since diploma, country of medical training, hours worked per week) and practice (urban vs rural location, solo vs group practice and participation in a network) characteristics were included, first in univariate analyses. Variables significant to a *p*-value less than 0.2 were retained for multivariate modelling using stepwise regression. Given the significant co-linearity between age and years since obtaining diploma, only years since obtaining diploma was retained for the multivariate model. Main predictors for engagement in complementary activities were presented through incident risk ratios (IRR), an IRR of 1.01 corresponding to 1% higher chance to be engaged in one additional complementary activity. Statistical analyses were performed in STATA (Version 14.0, Stata Corp, College Station, USA).

## Results

Of 600 GPs in Canton Vaud, 168 completed a survey (28%), 121 online and 47 using a paper form (see Table 1). Demographic characteristics concerning mean age, female gender proportion and mean hours worked per week were comparable to those published by the *Foederatio Medicorum Helveticorum* (FMH) yearly on physician’s demographics in Switzerland (comparison not shown) [11]. Six questionnaires out of 168 (3.5%) had missing demographic data. Further, 20% of positive responses did not specify the number of hours per month, and 22% did not specify whether they receive remuneration.

**Table 1:** Demographic distribution of GPs participating (*n* = 162)^a^

General characteristics	
Mean age – years (±SD)	54.3 (±9.2)
Female gender – N (%)	55 (32.7%)
Country of medical education Switzerland – N (%)	147 (87.5%)
Mean years since doctor’s diploma – years (±SD)	28.2 (±9.8)
Specific characteristics	
In solo practice – N (%)	48 (30.2%)
Physician’s office location rural - N [%]	48 (28.6%)
Mean number of hours worked per week - h (±SD)	42.7 (±13.3)
Median number of hours worked per week - h (IQR)	43 (31–50)
Physicians working full time^b^ - N (%)	84 (51.8%)
Member of a medical network – N (%)	61 (36.3%)

^a^Six participants excluded because of missing demographic data

^b^In Switzerland, 50 h of activity weekly are considered a 100% workload

**Fig. 1:**
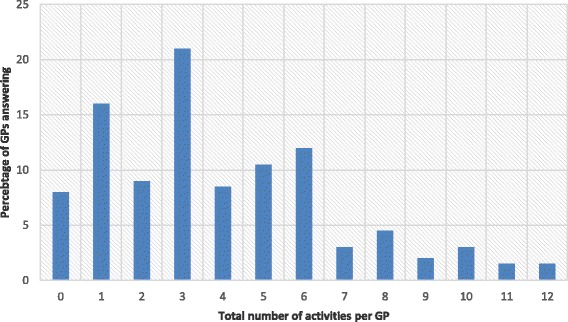
Total number of activities performed by general practitioner (GP), ranging from 0 to 12 (*N* = 162).^a^Six participants excluded because of missing demographic data

Overall, 92% (149) of GPs were engaged in at least one activity in addition to their standard consultations, 85% of respondents of the paper form and 94% of online participants, with as many as 12 activities per GP (Fig. 1). Overall, GPs spent 5.8 h per week on these activities and 83% of physicians were paid for at least one of their engagements (see Table 2). GPs were most implicated in community care giving (72% of GPs), but those engaged in teaching or care giving in specific structures (e.g. nursing homes) dedicated the most time (median > 12 h / month). When dividing the time dedicated per month by working days, GPs spent a daily average of 19 min on teaching and 20 min in specific external structures (e.g. nursing homes). Frequent responses in the field ‘other’ were participating in research projects, organizing quality circles and being members of medical or non-medical foundations.

**Table 2:** Number of, workload, remuneration and characteristic of participating GPs stratified by activity categories (*n* = 162)^a^

Category [question number]	Number of GPs (%)	Median hours per month spent on this activity	Number of GPs reporting at least one remunerated activity (% of GPs implicated)	Gender female	Mean age (±SD)	Practice in rural area
At least one activity [All questions: 1–17]	149 (92%)	23.4 h / mth	123 (83%)	47 (32%)	54.5 (±9.23)	45 (30%)
Teaching [1, 2]	83 (51%)	12.2 h / mth	74 (89%)	21 (38%)	55.2 (± 1.02)	27 (56%)
Care giving in specific structures [3–7]	90 (56%)	12.1 h / mth	74 (77%)	27 (49%)	55.1 (± 0.99)	32 (67%)
Medical expert or company doctor [8, 9]	66 (41%)	7.8 h / mth	52 (81%)	16 (29%)	55.0 (± 1.11)	24 (50%)
Community care giving [10–15]	117 (72%)	7.6 h / mth	73 (61%)	31 (58%)	54.6 (± 0.86)	38 (79%)
Other activities [16, 17]	41 (25%)	7.7 h / mth	29 (69%)	9 (16%)	57.1 (± 1.19)	12 (25%)

^a^Six participants excluded because of missing demographic data

The primary predictors of engagement in a greater number of complementary activities were working in a rural area (incident rate ratio (IRR) of 1.29 for higher number of complementary activities, 95% CI 1.05–1.57) and a greater weekly workload of standard consultations (IRR 1.01 for higher number of complementary activities per hour spent in-office, 95% CI 1.01–1.02, or IRR 1.14 for 10 additional worked hours, 95% CI 1.06–1.24). Female gender, years since obtaining medical diploma and being member of coordinated network were not associated with engagement in complementary activities in our multivariate analysis (see Table 3).

**Table 3:** Poisson regression model of physician characteristics associated with increasing number of activities outside regular practice (*N* = 162)^a^

Characteristic (N = 162)	Univariate IRR (95% CI)	*p*=	Multivariate IRR (95% CI)	*p*=
Gender (female) (*N* = 55)	0.66 (0.51–0.86)	0.002	0.80 (0.60–1.05)	0.11
Age (years)^b^	1.01 (1.00–1.02)	0.042	–	–
Years since obtaining medical diploma (years)	1.01 (1.00–1.02)	0.024	–	–
Trained in Switzerland (*N* = 147)	0.99 (0.69–1.42)	0.95	–	–
**Hours worked per week on in-office care giving**	1.02 (1.01–1.02)	< 0.001	**1.01 (1.01–1.02)**	**< 0.001**
**Practice in rural area (*N*** **= 48)**	1.32 (1.05–1.65)	0.015	**1.29 (1.05–1.57)**	**0.01**
Solo practice (N = 48)	0.94 (0.72–1.22)	0.63	–	–
Physician member of coordinated network (ie Delta network) (*N* = 61)	1.20 (0.96–1.49)	0.11	–	–

*IRR* Incident rate ratio. Statistically significant values in multivariate regression are shown in bold

^a^Six participants excluded because of missing demographic data

^b^Age not included in multivariate model despite being at *p* < 0.05 because of significant co-linearity with years since obtaining medical diploma

## Discussion

In this cross-sectional survey of GPs in Vaud, Switzerland, the vast majority of participants was engaged in one or more complementary activities and spent a considerable amount of time on these tasks. The primary predictor of participating in complementary activities, beyond working more hours per week on standard consultations, was practicing in a rural area, where other health care providers may be hard to reach [12].

As the shortage of GPs in Switzerland is predicted to be greatest rural areas [3], not only do these areas face a drop in the provision of basic medical services, but also potentially in diverse fields of health care delivery such as in-office teaching, nursing home care and other community activities. One possibility is that rural physicians participate in more complementary activities in order to increase their income. However, a study in Germany, which has a similar health-care system as Switzerland, showed nearly equal incomes of rural and urban GPs through standard in-office care, measured on the number of private insured patients [13]. Thus, remuneration of the complementary activities seems unlikely to be a major motivator.

In the current study, GPs reported spending an average of 19 min daily on teaching and 20 min in specific external structures (e.g. nursing homes), each corresponding to an average in-office consultation in Switzerland [7]. This is more than Gottschalk et al. reported for the USA in 2005 (13 and 5 min respectively) [14] but similar to findings from Granja et al. for Portugal in 2015 (15 min spent on teaching on average per day) [15], indicating that these health care areas may be relying more on GPs engagement over time. An increase in time spent on complementary activities would support Cohidon et al., who compared a more limited number of activities between 1993 and 2012 using a nation-wide sample of Swiss GPs and observed an increase from 28 to 66% of GPs involved in paid activities beyond their standard functions [7]. As in general, GPs tend to spend only about half their time on direct in-office patient contact [5], it is clear that out-of-practice medical services are taking an important role in their working days [16].

Given the number and variety of community roles filled by GPs, the new generation of medical professionals who value an equilibrated work-life balance and lighter weekly workload [17] may not be able to cover all needs. On the other hand, as complementary activities seem to strengthen the attractiveness of primary care [18, 19], GPs may favour complementary activities at the expense of in-office consultations. Regardless, the augmenting implication of GPs in these complementary activities can be explained either by an augmented need for GPs in out-of-practice medical care delivery or by the wish of primary physicians for a broader spectrum of work tasks.

Women appear to participate in fewer activities in univariate analyses (IRR 0.66 (95% CI 0.51–0.86)), however this result is no longer statistically significant when accounting for other physician and practice characteristics. This may be due to the observation that women in our study were younger than participating men, as age was significantly associated with more involvement in complementary activities. In the USA, spending more hours per week on standard consultations and working in a rural area were both independently associated with higher rates of participating in volunteer activities [8].

### Strengths and limitations

We had a relatively small sample size with a low response rate. As the study is based on self-reported data, possible declaration bias including recall bias cannot be excluded. Declaration bias including recall bias could result in GPs over- or underestimating their complementary engagement. As we are investigating the association between physician variables and engagement in complementary activities, differential recall bias seems unlikely to explain our primary results of an association between greater involvement and working in rural areas. Given our broad definition of complementary activities, it may not be surprising that most GPs are involved in at least one activity. Enabling participation through paper form during continuing medical education courses in addition to an online survey allowed us to achieve a more diverse sample, as GPs who filled out the paper form reported fewer complementary activities than GPs answering online. Further, while we asked about remuneration, GPs did not specify the exact amount. Important discrepancies between financial incentives perceived in the different categories could not be detected through this study. Our study shows interesting trends, but is from only one sample and should be confirmed using similar definitions in other settings.

## Conclusions

GPs provide not only excellent primary care in Switzerland [20], but as this study shows, play an important role in a multitude of educational, professional, and community activities. These findings support the importance of ensuring a steady supply of GPs. There are few existing studies that attempt to quantify these relevant aspects of complementary health care delivery, despite widespread reports of GP shortages. Presenting the broad spectrum of activities incorporated in primary care in the medical curriculum could help motivate young physicians to become GPs.

## Additional files

Additional file 1: Contains the english translation of the questionnaire used to provide information from GPs. (PDF 412 kb)

Additional file 2: Contains the original questionnaire in French language used to provide information from GPs. (PDF 243 kb)

## Abbreviations

CI: Confidence interval
FMH: Foederatio Medicorum Helveticorum (Swiss Physician’s Association)
GPs: General practitioners
IRR: Incident risk ratio
SD: Standard Deviation
US: United States (of America)

## References

[1] Pécoud A. De la pléthore à la pénurie de médecins : tentative de comprendre. Rev Med Suisse. 2016;89 17300090

[2] Waeber G, Pécoud A, Cornuz J, Gaspoz JM, Perrier A. Relève en médecine interne générale: enjeux et perspectives. Rev Med Suisse. 2009;188 19267050

[3] Weigel AM, Ullrich F, Shane DM, Mueller KJ (2016). Variation in primary care service patterns by rural-urban location. J Rur Health.

[4] Monnier M. Médecins de premier recours en Suisse romande: Qui sont-ils? Que font-ils? Prim Care. 2004;41

[5] Gilchrist V, McCord G, Schrop SL, King BD, McCormick KF, Oprandi AM (2005). Physician activities during time out of the examination room. Ann Fam Med.

[6] Jaccard Ruedin H, Weaver F (2009). Ageing workforce in an ageing society - Combien de professionnels de santé seront nécessaires en Suisse d’ici 2030?.

[7] Cohidon C, Cornuz J, Senn N (2015). Primary care in Switzerland: evolution of physicians' profile and activities in twenty years (1993-2012). BMC Fam Pract.

[8] Grande D, Armstrong K (2008). Community volunteerism of US physicians. J Gen Intern Med.

[9] Gruen RL, Campbell EG, Blumenthal D (2006). Public roles of US physicians: community participation, political involvement, and collective advocacy. JAMA.

[10] Pathman DE, Steiner BD, Williams E, Riggins T (1998). The four community dimensions of primary care practice. J Fam Pract.

[11] Hostettler S, Kraft E. FMH Aerztestatistik 2015. http://www.fmh.ch/files/pdf17/SAEZ_12-13_Artikel_Aerztestatistik_2015_D.pdf. Accessed 27.09.2017.

[12] Weinhold I, Gurtner S (2014). Understanding shortages of sufficient health care in rural areas. Health Policy.

[13] Steinhaeuser J, Joos S, Szecsenyi J, Miksch A (2011). A comparison of the workload of rural and urban primary care physicians in Germany: analysis of a questionnaire survey. BMC Fam Pract.

[14] Gottschalk A, Flocke SA (2005). Time spent in face-to-face patient care and work outside the examination room. Ann Fam Med.

[15] Granja M, Ponte C, Cavadas LF (2014). What keeps family physicians busy in Portugal? A multicentre observational study of work other than direct patient contacts. BMJ Open.

[16] Chen MA, Hollenberg JP, Michelen W, Peterson JC, Casalino LP (2011). Patient care outside of office visits: a primary care physician time study. J Gen Intern Med.

[17] Bickel MA, Brown AJ (2005). Generation X: implications for faculty recruitment and development in academic health centers. Acad Med.

[18] Le Floch B, Bastiaens H, Le reste YJ, Lingner H, Hoffman D, Czachowski S (2016). Which positive factors determine the GP satisfaction in clinical practice? A systematic literature review. BMC Fam Pract.

[19] Bitterman H, Vinker S (2014). Extending the boundaries of family medicine to perform manual procedures. Isr J Health Policy Res.

[20] OECD Reviews of Health Systems, Switzerland, 2011. http://www.oecd.org/els/health-systems/oecdreviewsofhealthsystems-switzerland.htm. Accessed 27 Sept 2017.

