# Supplementary material for: Participation in medical activities beyond standard consultations by Swiss general practitioners: a cross-sectional study
Source: BMC Fam Pract. 2018 May 3;19:52. doi: 10.1186/s12875-018-0738-1 (PMC5934800; doi:10.1186/s12875-018-0738-1)
Supplement: Supplementary file 1 — Contains the english translation of the questionnaire used to provide information from GPs. (PDF 412 kb) [file 12875_2018_738_MOESM1_ESM.pdf]

Médecins de famille Vaud
Association vaudoise des médecins de famille

FAMCOM STUDY

Policlinique Médicale Universitaire
CH-Lausanne

№ ____________

MEDICAL ACTIVITIES PERFORMED BEYOND
STANDARD CONSULTATIONS BY SWISS GENERAL PRACTITIONERS
(GENERAL / INTERNAL MEDICINE, FAMILY MEDICINE, PEDIATRICS)

Besides their usual in-office activities (patient consultations, documentation linked to consultations), many general practitioners perform additional medical activities. We would like to quantify your role in these activities in order to increase our understanding and emphasize the role of General Practitioners in our communities.

**Do you perform one or more of the following activities ?**	**NO**	**YES**	**If yes, please provide the mean number of hours per month spent on the activity (monthly average over a year), and if you get paid for the activity or not, without specifying the amount.**
1. Pre-graduate education (medical students, trainees)			How many hours per month? ____<br>Do you perceive monetary incentives? Yes ☐ No ☐
2. Post-graduate education (residents)			How many hours per month? ____<br>Do you perceive monetary incentives? Yes ☐ No ☐
3. School doctor			How many hours per month? ____<br>Do you perceive monetary incentives? Yes ☐ No ☐
4. Physician in a nursing home			How many hours per month? ____<br>Do you perceive monetary incentives? Yes ☐ No ☐
5. Advisor for home-care delivery (*Spitex* e.g.)			How many hours per month? ____<br>Do you perceive monetary incentives? Yes ☐ No ☐
6. Physician in a mobile program (palliative care, geriatrics, geriatric psychiatry)			How many hours per month? ____<br>Do you perceive monetary incentives? Yes ☐ No ☐
7. Physician for center for disabled adults			How many hours per month? ____<br>Do you perceive monetary incentives? Yes ☐ No ☐
8. Physician for a private company			How many hours per month? ____<br>Do you perceive monetary incentives? Yes ☐ No ☐
9. Physician expert for:			
A) Professional driving			How many hours per month? ____<br>Do you perceive monetary incentives? Yes ☐ No ☐
B) Firefighter			How many hours per month? ____<br>Do you perceive monetary incentives? Yes ☐ No ☐
C) Aviation			How many hours per month? ____<br>Do you perceive monetary incentives? Yes ☐ No ☐
D) Insurance			How many hours per month? ____<br>Do you perceive monetary incentives? Yes ☐ No ☐
E) Other : ____			How many hours per month? ____<br>Do you perceive monetary incentives? Yes ☐ No ☐

FAMCOM STUDY

№ ______

	NO	YES	If yes, please provide the mean average of hours per month spent on the activity (monthly average over a year), and if you get paid for the activity or not, without specifying the amount.
10. Physician for the Health and Migration Network (RESAMI)			How many hours per month? ______<br>Do you perceive monetary incentives? Yes ☐ No ☐
11. Prescribing methadone (consultation time)			How many hours per month? ______<br>Do you perceive monetary incentives? Yes ☐ No ☐
12. Community-based prevention (health promoting organizations, e.g.)			How many hours per month? ______<br>Do you perceive monetary incentives? Yes ☐ No ☐
13. Sport medicine			How many hours per month? ______<br>Do you perceive monetary incentives? Yes ☐ No ☐
14. Physician for events (concerts, sport events, e.g.)			How many hours per month? ______<br>Do you perceive monetary incentives? Yes ☐ No ☐
15. Political activities (professional societies included)			How many hours per month? ______<br>Do you perceive monetary incentives? Yes ☐ No ☐
16. Other : ______			How many hours per month? ______<br>Do you perceive monetary incentives? Yes ☐ No ☐

Do you participate in a managed care network (Delta network, e.g.) ? Yes ☐ No ☐
If yes, which one(s) ? ______

In which country was the majority of your medical education ?
Switzerland ☐
France ☐
Other (please specify) ☐ ______

How many physicians are there in your office, in addition to yourself ? ______

What percent of full time do you work each week (all medical activities included)? ______ %

On average, how many hours per week do you work as a general practitioner (not considering all the above mentioned additional activities, but including administrative tasks) ? ______ hours / week

In what type of area is your office located (e.g.) ?
Urban (Lausanne) ☐ **Peri-urban (Ecublens)** ☐ **Rural (Savigny)** ☐ **?**

Are you : female ☐ **or male** ☐ **?**

In what year were you been born ? ______

In what year did you get your medical diploma ? ______

Thank you very much !

Comments : ______
