# Supplementary material for: Participation in medical activities beyond standard consultations by Swiss general practitioners: a cross-sectional study
Source: BMC Fam Pract. 2018 May 3;19:52. doi: 10.1186/s12875-018-0738-1 (PMC5934800; doi:10.1186/s12875-018-0738-1)
Supplement: Supplementary file 2 — Contains the original questionnaire in French language used to provide information from GPs. (PDF 243 kb) [file 12875_2018_738_MOESM2_ESM.pdf]

Médecins de famille Vaud
Association vaudoise des médecins de famille

ETUDE FAMCOM

Policlinique Médicale Universitaire
CH-Lausanne

№ ______

ACTIVITÉS MÉDICALES ASSURÉES PAR LES MÉDECINS DE FAMILLE
EN PLUS DE LEURS CONSULTATIONS EN CABINET
(MÉDECINE GÉNÉRALE, MÉDECINE INTERNE, MÉDECINE INTERNE GÉNÉRALE, PÉDIATRIE)

A côté de leur activité en cabinet (consultation de patients, documentation liée à cette prise en charge, etc.), les médecins de famille pratiquent souvent d'autres activités. Nous aimerions recenser ces activités afin de mieux comprendre et de valoriser le rôle des médecins de famille.

**Pratiquez-vous l'une ou l'autre de ces activités ?**	**NON**	**OUI**	**Si oui, veuillez préciser le nombre d'heures par moi (moyenne mensuelle sur un an) et leur éventuelle rémunération financière, sans préciser le montant.**
1. Formation pré-graduée (étudiants en médecine, stagiaires)			Combien d'heures par mois? ______<br>Avez-vous une rémunération financière? Oui ☐ Non ☐
2. Formation post-graduée (médecins-assistants)			Combien d'heures par mois? ______<br>Avez-vous une rémunération financière? Oui ☐ Non ☐
3. Médecin scolaire			Combien d'heures par mois? ______<br>Avez-vous une rémunération financière? Oui ☐ Non ☐
4. Médecin en Etablissements Médico-Sociaux (EMS)			Combien d'heures par mois? ______<br>Avez-vous une rémunération financière? Oui ☐ Non ☐
5. Médecin-conseil pour les soins à domicile (CMS, Spitex, etc.)			Combien d'heures par mois? ______<br>Avez-vous une rémunération financière? Oui ☐ Non ☐
6. Médecin dans une équipe mobile (soins palliatifs, gériatrie, psycho-gériatrie, etc.)			Combien d'heures par mois? ______<br>Avez-vous une rémunération financière? Oui ☐ Non ☐
7. Médecin d'un centre socio-éducatif			Combien d'heures par mois? ______<br>Avez-vous une rémunération financière? Oui ☐ Non ☐
8. Médecin pour une entreprise			Combien d'heures par mois? ______<br>Avez-vous une rémunération financière? Oui ☐ Non ☐
9. Médecin conseil et expert			
A) Conduite professionnelle			Combien d'heures par mois? ______<br>Avez-vous une rémunération financière? Oui ☐ Non ☐
B) Sapeurs pompiers			Combien d'heures par mois? ______<br>Avez-vous une rémunération financière? Oui ☐ Non ☐
C) Aviation			Combien d'heures par mois? ______<br>Avez-vous une rémunération financière? Oui ☐ Non ☐
D) Assurance			Combien d'heures par mois? ______<br>Avez-vous une rémunération financière? Oui ☐ Non ☐
E) Autres : ______			Combien d'heures par mois? ______<br>Avez-vous une rémunération financière? Oui ☐ Non ☐

Médecins de famille Vaud
Association vaudoise des médecins de famille

ETUDE FAMCOM

Policlinique Médicale Universitaire
CH-Lausanne

№

	NON	OUI	Si oui, veuillez préciser le nombre d'heures par moi (moyenne mensuelle sur un an) et leur éventuelle rémunération financière, sans préciser le montant.
10. Médecin pour le Réseau de Santé et Migration (RESAMI)			Combien d'heures par mois? ___ Avez-vous une rémunération financière? Oui ☐ Non ☐
11. Médecin prescripteur de méthadone (temps de consultation)			Combien d'heures par mois? ___ Avez-vous une rémunération financière? Oui ☐ Non ☐
12. Activité de prévention communautaire (ligues de la santé, etc.)			Combien d'heures par mois? ___ Avez-vous une rémunération financière? Oui ☐ Non ☐
13. Médecin du sport			Combien d'heures par mois? ___ Avez-vous une rémunération financière? Oui ☐ Non ☐
14. Médecin dans des manifestations (concerts, réunions sportives, etc.)			Combien d'heures par mois? ___ Avez-vous une rémunération financière? Oui ☐ Non ☐
15. Activités politiques (sociétés professionnelles incluses)			Combien d'heures par mois? ___ Avez-vous une rémunération financière? Oui ☐ Non ☐
16. Autre : ___			Combien d'heures par mois? ___ Avez-vous une rémunération financière? Oui ☐ Non ☐

Appartenez-vous à un réseau de médecin (réseau Delta,….) ? Oui ☐ Non ☐
Si oui, lequel ou lesquels ? ___

Quel est votre pays de formation médicale ?
Suisse ☐
France ☐
Autre (veuillez spécifier) ☐ ___

Avec combien d'autres médecins partagez-vous votre cabinet (en plus de vous-même) ? ___

A quel pourcentage travaillez-vous (toutes activités médicales incluses) ? ___ %

Combien d'heures travaillez-vous par semaine (en moyenne) en tant que médecin de famille? (Hors emplois complémentaires mentionnés dans le tableau, mais tâches administratives comprises.) ___ heures / semaine

Votre cabinet est-il situé dans un milieu (p.ex.) :
urbain (Lausanne) ☐ **périurbain (Ecublens)** ☐ **rural (Savigny)** ☐ **?**

Êtes-vous : femme ☐ **ou homme** ☐ **?**

Quelle est votre année de naissance ? ___

Quelle est votre année de diplôme de médecin ? ___

Merci beaucoup !

Remarques : ___
